# Predicting Topology Propagation Messages in Mobile Ad Hoc Networks: The Value of History

**DOI:** 10.3390/s20010024

**Published:** 2019-12-19

**Authors:** Pere Millán, Carles Aliagas, Carlos Molina, Roc Meseguer, Sergio F. Ochoa, Rodrigo M. Santos

**Affiliations:** 1 Department of Computer Engineering and Mathematics, Universitat Rovira i Virgili, 43007 Tarragona, Spain; pere.millan@urv.cat (P.M.);; 2 Department of Computer Architecture, Universitat Politècnica de Catalunya, 08034 Barcelona, Spain; 3 Department of Computer Science, Universidad de Chile, Santiago 8370456, Chile; sochoa@dcc.uchile.cl; 4 Department of Electrical and Computers, Universidad Nacional del Sur, CONICET, Bahía Blanca 8000, Argentina

**Keywords:** network topology prediction messages, history-based prediction, mobile ad hoc networks, urban emergencies

## Abstract

The mobile ad hoc communication in highly dynamic scenarios, like urban evacuations or search-and-rescue processes, plays a key role in coordinating the activities performed by the participants. Particularly, counting on message routing enhances the communication capability among these actors. Given the high dynamism of these networks and their low bandwidth, having mechanisms to predict the network topology offers several potential advantages; e.g., to reduce the number of topology propagation messages delivered through the network, the consumption of resources in the nodes and the amount of redundant retransmissions. Most strategies reported in the literature to perform these predictions are limited to support high mobility, consume a large amount of resources or require training. In order to contribute towards addressing that challenge, this paper presents a history-based predictor (HBP), which is a prediction strategy based on the assumption that some topological changes in these networks have happened before in the past, therefore, the predictor can take advantage of these patterns following a simple and low-cost approach. The article extends a previous proposal of the authors and evaluates its impact in highly mobile scenarios through the implementation of a real predictor for the optimized link state routing (OLSR) protocol. The use of this predictor, named OLSR-HBP, shows a reduction of 40–55% of topology propagation messages compared to the regular OLSR protocol. Moreover, the use of this predictor has a low cost in terms of CPU and memory consumption, and it can also be used with other routing protocols.

## 1. Introduction

During an urban emergency, the regular communication infrastructure in the affected area tends to collapse, given the need of the people to know about family and friends potentially affected by the event. In these scenarios, the radio systems used by first response organizations (e.g., firefighters, police and medical personnel) for the coordination of their activities in, and between, their teams, are limited in managing multiple communication channels and in the delivery of digital information [1,2,3]. Therefore, digital communication is required to increase the interaction capability among first responders in the work area. These communication limitations have frequently been addressed using mobile ad hoc or opportunistic networks, as well as routing protocols on these networks [4,5,6]. These networks have low bandwidth and their topology is highly dynamic, and as such, they require the exchange of an important number of topology propagation (TP) messages to keep the delivery of regular messages under control. The exchange of TP messages helps reduce the probability of overflowing the network with regular messages that do not reach the destination, rendering the communication system useless at supporting the coordination of the emergency response activities. Provided that many of these communication systems involve mobile ad hoc networks, the routing protocols used to support interactions among nodes (i.e., the first responders) must be simple, efficient and reliable and have the capability of quickly adapting themselves to changes in the network topology [7,8,9,10]. Thus, these protocols intend to maximize the reachability of the target nodes, by consuming as little energy from the network as possible.

Regardless of the extensive research done in the area, addressing this communication challenge, considering the previous constraints, is still an open issue. Some recent proposals try to deal with this challenge using deep learning in static or quasi-static networks [11]. Although deep learning techniques have shown positive results, these approaches are quite limited when used in mobile scenarios, like emergency responses. Moreover, they require a previous training stage to generate the network model. Novel solutions are therefore required to reduce the number of TP messages exchanged among the network nodes, and consequently, decrease the network traffic and energy consumption on the participating devices. The potential solutions should address this challenge, while keeping the routing capability of the used protocol.

In a previous work the authors proposed a history-based approach to help address the stated communication challenge [12]; however, the validation of such a proposal was based on an analysis of the number of TP messages that can be potentially predicted by such a mechanism. This paper extends that previous work by presenting the implementation of a real predictor, and also by presenting a study that evaluates its prediction performance in highly mobile scenarios—this shows the feasibility of using the proposal in practice. Moreover, this article presents two prediction algorithms, one for each type of node involved in the propagation of the network topology information.

The proposed history-based predictor (HBP) acts as a transparent communication intermediary, located between the routing and network layers, therefore, no changes to routing protocols are required for using it. In order to quantify its benefits, the predictor was implemented over the optimized link state routing (OLSR) protocol. The resulting implementation, named OLSR-HBP, was evaluated using simulations that involved several mobile scenarios. The obtained results were highly positive, showing that this prediction approach represents a tangible alternative to reduce traffic and energy consumption generated by the exchange of topology propagation messages.

The rest of the paper is organized as follows. Section 2 presents the related work on message prediction in computer networks. Section 3 introduces the conceptual aspects of the prediction proposal. Section 4 describes the implementation of the OLSR-HBP and presents the validation of such an implementation using a static interaction scenario, since it allows for determining if the proposal is working or not according to what is expected of it. Section 5 presents and discusses the experimental results of using OLSR-HBP in mobile scenarios considering that is the target of this proposal. Section 6 summarizes the conclusions and future work.

## 2. Related Work

The overall performance of a routing protocol can significantly improve when using a prediction mechanism that has a high percentage of success. However, the use of predictors increases the complexity of the routing protocols, because of the additional hardware and software required to make and validate predictions. Moreover, penalty mechanisms are usually introduced to the system when there is a high rate of mispredictions, which negatively affect the performance of these protocols.

Prediction mechanisms have been embedded in routing protocols to foresee several aspects of a network, such as nodes mobility [13], reliability of its topology [14,15] and quality of links and end-to-end paths [16,17,18,19,20,21,22,23,24]. These mechanisms have also been used to reach particular communication goals, e.g., to support message delivery in mobile ad hoc networks (MANETs) [25,26,27,28], reduce the traffic [29,30,31] and keep the energy consumption in these infrastructures under control [32,33,34].

Prediction strategies have been used to predict nodes mobility in MANETs; for instance, Rosa et al. [15] developed a technique to foresee disconnection of nodes in these networks. Their approach is based on the Markov chain model, and it considers topology information and global mobility to indicate possible disconnections in the near future. Su et al. [13] assumed a local mobility approach to estimate the link expiration time between adjacent mobile nodes. This prediction helps reconstruct routes proactively, and therefore, forecast a fairly accurate topology for the network. Another local mobility prediction approach was proposed by Chen et al. [14] to foresee movements of nodes, from its current position to the next location, based on recent history. This prediction approach facilitates making decisions on route maintenance and resource reservation.

When prediction is used to support routing in MANETs, two main approaches arise: (1) dealing with classical routing algorithms but supported by metric-based prediction; and (2) dealing with algorithms based on machine learning or deep learning. Assuming the former approach, Azzouni et al. [25] proposed a dynamic routing framework that deals with real-time traffic to understand the traffic behaviour, and allows generating message forwarding rules that are stored into the routing tables. Sendra et al. [26] introduced a mechanism that uses artificial intelligence to improve the performance of routing protocols. This mechanism chooses the best data transmission paths according to the best criteria and based on the network status. Finally, Lin et al. [27] proposed a Quality of Service (QoS) aware adaptive routing method to enable adaptive packet forwarding, by applying reinforcement learning in multi-layer hierarchical software-defined network.

In the latter approach, classical routing algorithms are considered, but they involve the use of metrics based on machine learning to make predictions. For instance, Azzouni et al. [28] proposed a method that builds the network traffic matrix in real-time, by means of time series prediction, achieving high prediction accuracy in a very short training time.

Concerning the prediction of topology propagation (TP) messages based on historical information, in previous works, the authors have shown that the traffic generated by a link-state routing protocol, like OLSR [35], grows almost exponentially with the number of nodes when different node densities are considered in the analysis [29,30]. Therefore, a network with a large number of nodes requires the exchange of a huge amount of TP messages to inform the nodes about the topology changes. These messages not only overload the communication links, but also increase the energy consumption of the nodes. The authors have also shown that the problem of delivering so much control information through the network can be addressed using predictions [29,31]. Particularly, the OLSRp protocol was proposed to eliminate redundant control information, and thus, reduce CPU and energy consumption in MANETs. This prediction mechanism is based on the assumption that the last TP message sent by a node will probably be repeated during the next round of information delivery. Such a proposal has shown positive potential results in a simplified communication scenario, but its real capabilities need to be determined through more complex scenarios and real scenarios that consider the diversity of variables affecting the TP message exchange in MANETs.

Concerning end-to-end/link quality prediction, there have been several authors that address this topic. Typically, quality tracking [16,17,18] has been applied to select links with higher quality, and thus maximize the message delivery rate and minimize traffic congestion. Recently, time-series analysis has been considered to estimate link quality (LQ) and end-to-end quality (EtEQ) in the routing layer, involving real-world wireless mesh community networks. For instance, Millan et al. [19,20,21,22] shown that time-series analysis can be used to improve the performance of the routing protocol, by providing information that allows making appropriate and timely decisions. This contributes to maximize the message delivery rate and minimize traffic congestion at both levels (i.e., LQ and EtEQ) with a small average mean absolute error. Additionally, Bote-Lorenzo et al. [23] identified several problems and limitations of the predictive models used in [20], through the use of batch machine learning algorithms. Similarly, Lowrance and Lauf [24] provide an exhaustive study of the existing prediction methods to estimate link quality, considering online learning algorithms that increasingly update the network model.

Concerning energy consumption, Kim et al. [32] proposed a new metric that evaluates the capacity of batteries to estimate the future lifetime of nodes in order to determine which nodes can be part of an active route. Luo et al. [33] dealt with mobile prediction to extend the lifetime of nodes in an energy-efficient way. Finally, Shaihh et al. [34] focused on managing the limited battery capacity of nodes in a wireless sensor networks. Such a work reviewed several techniques to address this issue, including some of them that applies a kind of prediction.

Recently, cooperative network synchronization and cooperative network localization are becoming increasingly popular in wireless networks, as a way of improving the accuracy of clock synchronization and location information. For instance, Xiong et al. [36] developed a framework that analyses the performance limits of cooperative network synchronization, as a way to address the drawbacks that introduce traditional synchronization techniques in wireless networks. Wymeersch et al. [37] provided an overview of cooperative localization algorithms and applied them to ultrawide bandwidth wireless networks. Finally, Yuan et al. [38] focused on passive localization of asynchronous receivers in wireless sensor networks. The history-based prediction mechanism proposed in this article can be easily adapted to fit with these techniques, and thus help predict synchronization and location information.

## 3. History-Based Prediction Approach

This section describes the basis of our prediction approach, the dynamics of the prediction process as well as the the use of confidence mechanisms, the effects of dealing with different historical data-window sizes and finally, the potential benefits of the history-based prediction approach.

### 3.1. Basis

Link state routing protocols are typically used to support mobile applications in ad hoc interaction environments. These protocols, e.g., optimized link state routing protocol (OLSR) or open shortest path first (OSPF), use two types of messages to establish the current topology of the network and the quality of their links. Particularly, HELLO messages are used to detect the state and quality of adjacent links, and topology propagation (TP) messages to inform the current links to the rest of the nodes. This proposal focuses on predicting only the TP messages using a history-based approach.

**Figure 1:**
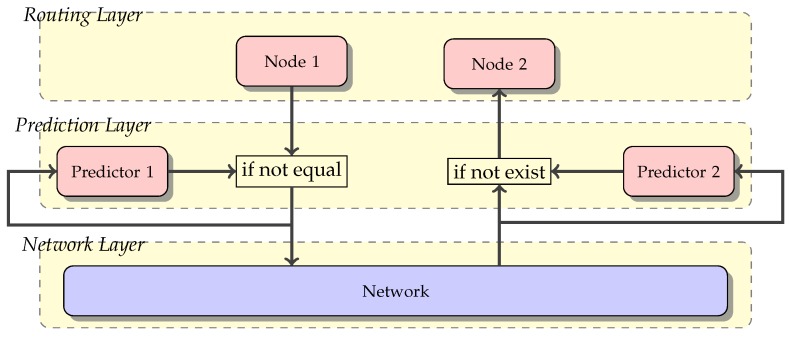
System architecture that includes the twin predictors.

In this approach, each node in charge of communicating a topology change in the network (e.g., Node 1 in Figure 1) must determine if the TP message to be sent can be predicted by the destination (e.g., Node 2), and as a result, the message transmission could be prevented. Consequently, when a TP message expected at the destination is not received, a prediction is made by such a node (e.g., Node 2) to generate the "missing" TP message. This message is passed to the upper routing layer as if the TP message had actually been received through the network. Thus, the source node reduces the amount of TP messages transmitted through the network without modifying the signalling information, and its processing by the destination nodes.

As shown in Figure 1, this prediction approach requires the participation of twin predictors (Predictors 1 and 2), which must have a high rate of accurate predictions in order to reduce the number messages delivered through the network. This prediction process is based on the fact that some topological states of the network have already appeared in the past; therefore, they can be predicted. In order to do that, each node keeps a table with the recent history of the TP messages requested to or received from its neighbours. When a prediction process is required, past topology information is used as input to determine the future topology of the network. Particularly, unbounded tables are used to store this information, since these structures provide flexibility to identify the mobility patterns of the nodes, and thus, to determine the next evolution of the network topology.

When required, Node 1 generates a TP message and Predictor 1 predicts the same message using historical information (Figure 1). If both messages are equal, it means that the TP message can be predicted by the destination (Node 2); therefore, such a message is not sent through the network. Algorithm 1 shows the operation of Predictor 1 at the nodes where TP messages originate.

The algorithm assumes that every TP message can be predicted by all neighbour nodes; i.e., generated by the routing layer. In order to be sure that such a prediction is feasible, the sender node checks that all current neighbours will be able to correctly generate the TP message (TPg) at destination. Therefore, the algorithm uses its $neighbourHistory_{i}$ to make a prediction for every neighbour node. If a neighbour is not able to make a right prediction, then the TPg message should be broadcast to neighbours through the network layer. In other cases, the TPg message is not broadcast (i.e., it is omitted). Additionally, the TPg message should be appended to every $neighbourHistory_{i}$.
**Algorithm 1:** Predictor 1 (TP msg sender) at origin node
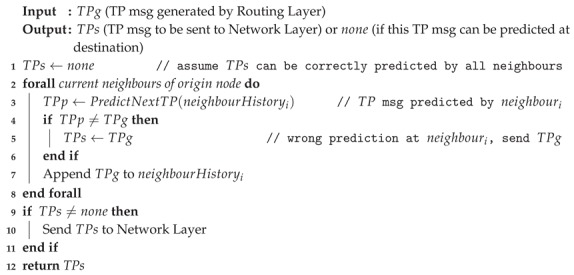


Consequently, if the predictor in Node 2 (i.e., the destination node) does not receive the TP message, it assumes that such a message can be predicted using the historical information. Therefore, it generates the message and passes it to Node 2. Thus, the destination node regularly receives the TP message and processes it, regardless of whether or not it was generated by the source node or local predictor. Working in such a way, this prediction approach becomes transparent for the routing protocols that use it. Algorithm 2 shows the operation of Predictor 2 at the nodes where TP messages would be received or generated by the predictor.

Periodically, a new TP message must be received by the destination node. When the time for the next TP message reception is over and no message was received, a new TP message is locally generated using the predictor and the corresponding TP history. Whether the TP message is received or generated, it is appended to the history and injected into the routing layer.

In the next section we present an example scenario that allows us to illustrate the dynamic of the prediction process and its potential benefits using confidence mechanisms and different sizes for the historical data-window.
**Algorithm 2:** Predictor 2 (TP msg receiver) at neighbour nodes
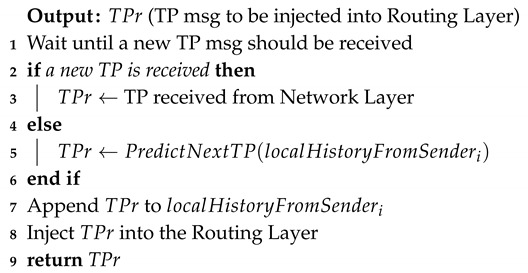


### 3.2. Dynamics of the Prediction Process

**Figure 2:**
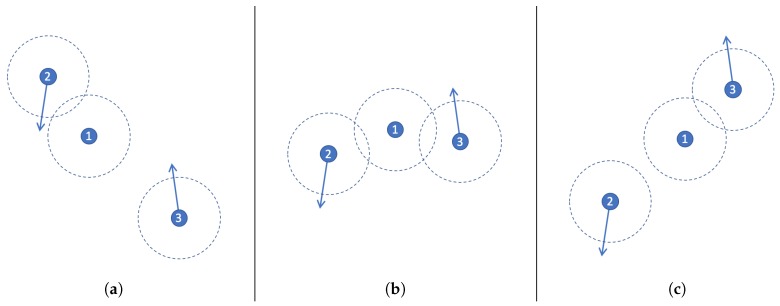
Example of a mobile scenario that involves three nodes, (**a**) First position, where nodes 1 and 2 can communicate, (**b**) Next position, where the 3 nodes can communicate, (**c**) Last position of nodes, where nodes 1 and 3 can communicate.

In order to illustrate the dynamic of the prediction process, let us consider the example shown in Figure 2 that involves two mobile and one stationary node. The direction in which the nodes are moving is represented with an arrow, and their communication threshold is indicated with a dashed circle.

The networking scenario depicted in Figure 2a indicates that communication between Nodes 1 and 2 is possible since their communication thresholds are overlapped; therefore, Node 1 can exchange TP messages with Node 2; however, they cannot exchange messages with Node 3. Then, such networking scenario evolves toward the topology depicted in Figure 2b, where Node 1 can communicate with both Nodes 2 and 3. Finally, in the last scenario (Figure 2c) the communication is possible between Nodes 1 and 3, but Node 2 is out of reach.

Each TP message has partial information about the current network topology, e.g., each node exchanges the list of neighbour nodes located to one-hop of distance. These messages are delivered with a certain frequency to communicate changes of the network structure. For instance, Node 1 in Figure 2a will send the following TP message while the network keeps such a topology: $NodeList_{Node1}=\left[2\right]$. In the scenario shown in Figure 2b the messages will be: $NodeList_{Node1}=\left[2,3\right]$. A possible sequence of TP messages for Node 1 starting in scenario 2a and finishing in 2c is the following: [2] [2] [2] [2] [2,3] [2,3] [2,3] [2,3] [3] [3] [3] [3].

The prediction process is conducted every time that a TP message should be delivered by a node, and it considers as input the mobility patterns of the neighbours of such a node. Each mobility pattern corresponds to a sequence of TP messages (one or more) that the node making the prediction has seen in the past and registered in its local table; therefore, the pattern can be identified analysing the local history table of that node. The history table also records supporting information that helps predict the next TP message when a mobility pattern is detected by a node. This information includes both, the most frequent TP message delivered after a certain mobility pattern was observed, and also the TP message delivered the last time that a particular pattern was identified. Summarizing, an entry of the history table will be composed by the following items:

- A mobility pattern represented by one or more TP messages;
- The potential next TP messages to be delivered (or predicted), which are represented by the list of TP messages that appeared after each particular mobility pattern;
- The supporting information that helps predict the output, i.e., the next TP message.

Table 1 shows an example of the history table of Node 1 considering the previous example. It assumes patterns with two consecutive TP messages, which are shown in the first column of the table. The second column indicates all TP messages that have appeared after the pattern, and the third column records the number of times that these TP messages have appeared in the past. Finally, the fourth column indicates if the TP message has been the last one observed after the pattern identification.

Using this information we can define several strategies to predict the next TP message. Particularly, in this study we analyse the prediction performance of three different versions of the history-based prediction approach (HBP), which emphasizes the *last-value* (last column of the table), *most-frequent value* (third column) and *random value* (it uses a randomly selected message).

### 3.3. Using Confidence Mechanisms in the Prediction Process

The proposed prediction method includes a confidence mechanism to determine the likelihood of a right prediction. The purpose of this mechanism is to avoid incorrect predictions, which can skew the network topology map and decrease the reliability of the process. The confidence mechanism determines if each TP message had already been predicted in the past. If so, a counter is incremented by 1; otherwise, it is decremented by 1.

The counter is initialized to 1, and it can take values in the range from 0 to 3. A prediction is considered to have a high confidence value (i.e., high certainty of a right prediction) if the counter is equal to or higher than 2.

In order to determine the granularity of the historical information that is appropriate for conducting this prediction process, the HBP approach defines the *history depth* (HD) metric. This metric can be computed as the number of TP messages that composes a mobility pattern. For instance, if we consider a table with HD = 1, the number of messages that identifies each pattern is one, and there will be one entry in the table for every TP message appeared in the past. Considering this metric, it is reasonable to assume that a high HD leads to high prediction accuracy, but low prediction opportunities, because the message sequences are longer.

Although applying a confidence mechanism based on HD can improve the prediction accuracy, the opportunities of performing appropriate predictions are fixed if we consider a fixed HD (i.e., a fixed length for the sequence that represents the patterns). Therefore, in this HBP approach we use a *prediction tree* in which the HD is not initially fixed. In this tree the maximum HD is assumed to make a prediction. If it is not possible to make such an assumption (e.g., because there is no entry in the table or there is not enough certainty in the prediction), the HD will be decreased by 1. The predictor then attempts to make a new prediction, but using a shorter pattern. This will be repeated until the HD metric reaches 0.

The performance of the HBP approach was analysed with and without a confidence mechanism. To understand the limits of HBP and determine how far a particular version of this approach is from the best performance, different metrics were analysed. The repetition of TP messages over time was quantified, as well as the maximum prediction accuracy of the different HBP versions. Moreover, the number of incorrect predictions (for various HD values) that could be correctly predicted were also quantified. We assume that if a certain TP message has appeared in the past at least once, it could be correctly predicted. We also analysed the representativeness of the most-frequent messages with respect to the whole set of messages received by a node over time. This helps us understand the difficulty in making right predictions and let us know how much data (historical information) must be tracked to be able to make these predictions.

### 3.4. Using Different Historical Data-Window Sizes in the Prediction Process

This section presents an analysis that determines the effect of using different historical data-window sizes (HDWS), i.e., different amounts of historical data in the prediction process. The analysis involved HDWS of 1, 4 and 24 h. This means that the historical data used by the predictor was reset every 1 h, 4 h and 24 h respectively.

**Figure 3:**
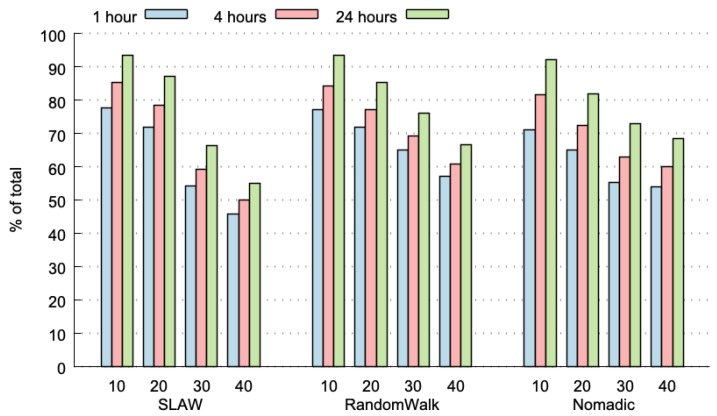
Predictability limit for different historical data-window sizes (HDWS) and node mobility patterns.

The experiments were performed during 24 h. Then, we split this historical data in 6 fragments of 4 h each, and also in 24 fragments of 1 h each. The results of every individual fragment were analysed and the average values of every fragment was computed. Figure 3 plots the predictability limit (percentage of TC messages that have already appeared in the past) for the three considered HDWS (i.e., 1, 4 and 24 h), with three different mobility models for the nodes (i.e., self-similar least action walk (SLAW), RandomWalk and Nomadic), and for 10 to 40 nodes participating in the network. The predictability limits range from 50% to more than 90%, with very low variance (average variance: 0.024% and maximum variance: 0.108%). As expected, the predictability limit increases with a larger HDWS; particularly, a HDWS of 24 h achieves the highest predictability limit, and a HDWS of 1 h presents the lowest values. These results can be explained, because in a 24 h time-period, there is a higher opportunity to find TP messages that have already appeared in the past.

**Figure 4:**
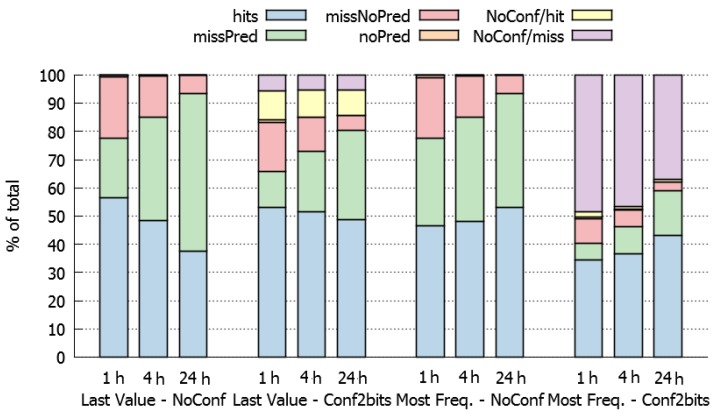
History-based prediction using a self-similar least action walk (SLAW) mobility model with 10 nodes, and last-value and most-frequent policies, with and without confidence (1 h/4 h/24 h).

To analyse the effect of using different HDWS more in-depth, we conducted a prediction process considering two prediction algorithms (particularly, the “last-value” and “most-frequent” approaches), with and without a confidence mechanism (particularly, with 2 bit confidence and no-confidence). The rest of the parameters assumed for the experiment were: SLAW mobility model, 10 nodes network and dynamic HBP (tree) with a maximum history-depth of 5. Figure 4 presents the results obtained. The average and maximum variance of these values are 0.038% and 0.274% respectively, which indicates a low data dispersion.

According to these results, when using a last-value prediction policy (left half of Figure 4), the HDWS should be short (1 h), because larger data-windows (4 and 24 h) decrease the percentage of hits and increase the percentage of total misses. This behaviour can be explained because larger HDWS have more variability and fewer opportunities to repeat last values.

On the other side, when using a most-frequent prediction policy (right half of Figure 4), the HDWS should be large (24 h) since it increases the percentage of hits. This result is coherent because in large HDWS there are more opportunities to find the sequence of TP messages that we are looking for to perform the prediction.

Concerning the use a confidence method to support the predictions, the aim is to reduce the number of bad predictions (misses) due to low confidence. The results show (second and fourth quarter in Figure 4) that the percentage of hits tends to decrease in these cases. The predictions that are not performed due to lack of confidence and would be misses (purple values in Figure 4), and are much larger for the most-frequent than for the last-value approach. The use of confidence jointly with the last-value approach is counterproductive, because most predictions that are not performed would have been hits (yellow values in Figure 4).

Finally, it is important to determine if it is better to increase the percentage of hits at the cost of a higher number of misses when confidence is not applied, or whether it is better to decrease the percentage of misses, and also the percentage of hits, applying confidence in the prediction. In a real scenario, this will depend on the time saved with the hits, the additional time spent with the misses and the time consumed when no prediction is made because of lack of confidence.

### 3.5. Potential Benefits of the HBP Approach

In a previous work the authors determined a reference number of TP messages that could be predicted using an HBP approach [12] considering simple and theoretical interaction scenarios. Such a research work shows that between 50% and 80% of the time, the TP messages to be predicted had already appeared in the past, and therefore they were potentially predictable. According to these preliminary results, the performance of the HBP approach does not depend on the mobility model used by the nodes or their speed, however, its prediction performance decreases, while increasing the node density in the network. These numbers establish the upper-bound limit achievable by this prediction approach.

In order to extend our understanding about the potential impact of the HBP approach we conducted new simulations, but using a real predictor implemented over OLSR protocol. The simulations involved more complex mobile scenarios.

## 4. Implementation of the OLSR-HBP

This section describes the implementation of the OLSR-HBP, the dynamic of the TP message prediction and the validation of the predictor implementation. Next we explain these three aspects of the proposal.

### 4.1. Implementation Aspects

As mentioned before, the predictor was implemented over the OLSR (optimized link state routing) protocol [35], a well-known proactive link state routing algorithm. When using OLSR, the network nodes periodically exchange routing information to keep a map of the network topology using HELLO and topology propagation (TP) messages. HELLO messages allow each node to discover its neighbouring nodes and obtain information about the state of the links with them, and TP messages allow multi-point relay (MPR) nodes to disseminate neighbour information throughout the network. The MPR nodes are the network nodes selected for propagating the topology information.

The implementation of the predictor, named OLSR-HBP, addresses the prediction of TP messages through an extension to the C++ code of the NS-3 OLSR module. NS-3 is a simulation tool that allows modelling networking scenarios, collecting statistics, defining initial network topologies, configuring wireless network interfaces and setting the mobility patterns of the nodes [39].

In order to make a prediction, OLSR-HBP analyses the historical information, recorded through sequences of TP messages, at both origin and destination nodes. Origin nodes (i.e., the MPR nodes) broadcast TP messages to different destination nodes. Due to the nodes mobility, the topology information changes in an independent way, therefore it is necessary to keep the sequences of TP messages separated for each pair of origin-destination nodes, and the prediction should be also made for each separated sequence. Every node should keep the sequences of TP messages updated for the nodes that receive these messages from it, and also for the nodes that send TP messages to it.

At the origin, the OLSR-HBP considers that the next TP message can be omitted (not transmitted) if all destination nodes can predict correctly (hit) such a TP message from their corresponding local information of TP sequences; we call this prediction situation a *global hit*. We have added data structures to the NS-3 OLSR module to keep the information of the individual sequences of TP messages for every pair of origin-destination nodes that interchange TP messages. OLSR-HBP uses these data structures to detect *global hits* at origin nodes and therefore, to prevent broadcasting this TP message.

At destination nodes, the predictor uses a timer to determine the potential arrival of TP messages from every possible origin node. If the timer expires and its expected TP message is not received, the destination node considers that the next TP message is a hit, therefore it can be predicted. In that case, a new message is generated by the predictor and processed as if it had been received from the origin. This information is also used to update the local topology historical information.

TP messages contain diverse information, particularly the IPv4 addresses of the originator node and their neighbours, the advertised neighbour sequence number (ANSN—a number that is incremented every time the topology information of the node changes), and the message sequence number (MSN—incremented every time a node sends a new control message).

When predicting a new TP message at the destination node, all this information should be generated. The address of the MPR nodes and the list of neighbours are generated using the historical information stored in the local tables of the nodes. To generate the ANSN, we use the last ANSN value received from the origin node. To produce MSNs, we introduced “gaps” in the sequence of MSNs, so that the destination nodes can detect these “gaps” and use them as MSN for the generated TP. If the last MSN received is not consecutive to the previous one, a gap is detected and the missing MSN is put in a buffer. When a new TP message is generated, its MSN value is taken from this buffer. If the buffer is empty, then the predictor uses an MSN value equal to 0.

In case of some transmission delay, destination nodes should wait extra time for a new TP message to arrive. When the TP message (received or predicted) is forwarded, the extra delay causes it to not combine with other pending OLSR messages in the same communication packet; it increases the number of packets transmitted (with just one TP message) and also the energy consumption in the network. To alleviate this problem we tuned the jitter parameter. The jitter is the additional time to wait before transmitting the TP message, in order to combine other later messages into the same packet. OLSR uses jitter values of 0–0.5 s. We have extended the OLSR-HBP jitter to increase opportunities to combine several messages into the same packet.

Despite the potential benefits of the OLSR-HBP, there are also a couple of potential limitations: (1) if a TP message that had been broadcast is lost during transmission, OLSR-HBP at the destination will wrongly assume that it was a *global hit* and thus, a wrong TP message will be generated; and (2) the predictor is not 100% accurate due to the delay in updating the routing information at the destination—the delay is caused by the extra time required for a new TP message to arrive from the origin node before a prediction can be made.

### 4.2. Analysis of the OLSR-HBP Implementation

The implementation of OLSR-HBP was validated using stationary network scenarios, since it allows us to be sure that the predictor is working as expected. The obtained results were analyzed using various network metrics. In these interaction scenarios we also compared the impact of using OLSR-HBP and the standard OLSR routing protocol. Results from this comparison establish a benchmark that explains the performance of OLSR-HBP in mobile interaction scenarios, which is the target of this proposal.

The simulation scenario considered a 4×4 wireless mesh backbone network, where edge routers generate packets for other edge routers, and inner routers just forward packets. This scenario is similar to the one described in [11]. Aligned with such an experiment we also considered 1 KByte ping data packets, sent every 1 and 10 seconds, which corresponds to a range from 1.2 to 12.2 Mbps of data traffic. The setting details are specified in Table 2.

With the NS-3 WiFi configuration, the coverage range of the nodes is approximately 70 m. It is due to the transmission gain (dB) and the energy threshold (dbm) required to allow the PHY layer of NS-3 to detect the signal. The simulations used the default configuration of the OLSR protocol [35]. This configuration considers the delivery of HELLO messages every two seconds and TP messages every five seconds.

Table 3 summarizes the main results of this study. In the considered static scenario, OLSR-HBP outperforms the OLSR routing protocol in several performance metrics. Moreover, the number of TP messages that can be correctly predicted (and thus not transmitted) achieves very high values (more than 99%), which indicates that the implementation of the predictor is working as expected.

In order to make a more in depth validation of the OLSR-HBP performance, and considering OLSR as ground truth, we compared both protocols considering the following performance metrics: precision, recall, F1 score, and area under the curve (AUC). Precision is the number of true positives (TrP) divided by number of TrP and false positives (FaP); thus precision is defined as follows: P=TrP/(TrP+FaP). Recall is the number of TrP divided by the number of TrP and false negatives (FaN); it means that R=TrP/(TrP+FaN). The F1 score is the harmonic mean of Precision and Recall. Finally, the AUC score is calculated by plotting the Recall against the false positive rate at various threshold settings. As OLSR-HBP is a multiclass predictor (it has as many classes as TC identifiers), we assumed the *micro* and *macro* averaging approaches to aggregate the metric scores of each class. Meanwhile the first approach considers the global counting of the total true positives, the second one calculates the metric for each class and find their unweighted mean.

Table 4 shows the performance results for the static scenario considering different packet generation periods. As expected, these metric results confirm that the proposed predictor is working properly. Therefore, the next step is to evaluate its performance in mobile scenarios to determine the percentage of message reduction that can be expected when it is used.

## 5. Evaluation of OLSR-HBP in Mobile Scenarios

In order to present the evaluation of OLSR-HBP in mobile scenarios, we first describe the experimental framework used in the simulations and the scenarios involved on it. Then, we present the results obtained by the predictor in the previous settings, and particularly analyse the control traffic decrease and memory overhead. Finally, we determine the actual impact of using this predictor in mobile networks considering the data transfer capabilities and also the energy consumption in the network. This impact was determined comparing the network performance when using the regular OLSR protocol, and then adding it the proposed predictor.

### 5.1. Experimental Framework

The performance of OLSR-HBP was analysed and compared using several settings and parameters. For each specific set of parameters, we executed ten variants of the tests, and then we computed the average, standard deviation and the minimum and maximum values of the observed variables. The parameters used in the simulations are specified in Table 5.

The deployment area, the number of nodes participating in the network and their mobility pattern was set simulating a regular (small) urban emergency, like a fire in a building or the partial collapse of a civil infrastructure. In these scenarios the incident commander (who is in charge of the emergency response process) and the team leaders represents the network nodes. Most of them have high mobility in the affected area following a self-similar least action walk (SLAW) pattern. Regarding the confidence, we performed various initial tests, first with 0 bits (no confidence) and then with confidence with 2 bits in order to determine an appropriate value for the simulations. After these tests we discarded the latter case (i.e., the confidence with 2 bits), and the reason was that the small number of errors produced at destination nodes, when no confidence was applied (about 0.5% of the TP messages predicted/generated at destination), were “amplified" by the confidence mechanism. This mechanism produced 40 times more wrong predictions at destination and 42% less TP messages omitted (not sent) at origin; therefore, the confidence was set in 0. Finally, the data traffic and the ping periods were set according to the recommendations given in [11].

### 5.2. Validation of OLSR-HBP

Similar to the prediction performance validation conducted for the static scenario, in this section we present the prediction performance of the model in a mobile scenario. Again, we assumed OLSR as ground truth and calculated precision, recall, F1 score and AUC as standard performance classification metrics. Table 6 summarizes the range of metrics values obtained when considering scenarios with 10 and 20 nodes, historical data-window sizes (HDWS) of 1 h and 4 h and also prediction with and without confidence. Moreover, data packet generation periods of 10, 1 and 0.1 s have been considered for all the simulations, as well as no data packet generation at all (shown in the first column of the table). The specific data of each simulation can be found in Appendix A.

In general, values range from 0.71 to 0.96, in the simulation with 10 nodes, 1 h HDWS and 2 bits of confidence being the one with better results; meanwhile the simulation with 20 nodes, 4 h HDWS and no confidence bits is the one with moderate results. Particularly, the AUC metric achieves the best results of the considered validation metrics, with values ranging from 0.89 to 0.95. In any case, all of the validation values can be considered as excellent or good results, and therefore, they demonstrate the effectiveness of the prediction model.

### 5.3. Performance of OLSR-HBP

The performance of the implemented predictor was evaluated considering the control traffic decrease and also the memory overhead. These network variables can be positively impacted by the proposed predictor and thus to improve the communication support in highly dynamic interaction scenarios, like urban emergency response processes. Next we describe these evaluations in detail.

#### 5.3.1. Control Traffic Decrease

Decreasing the signalling overhead (number of TP messages sent through the network) is an important goal for this proposal. As explained before, the routing protocol that is used can avoid delivering a TP broadcast when all the destination nodes are able to correctly predict it (i.e., it is a *global hit*). If only one of the destination nodes is unable to predict the TP message, it has to be broadcast. This requirement of a global hit will produce results that are worse than those presented in Section 4, where the hit was considered just at the origin node.

**Figure 5:**
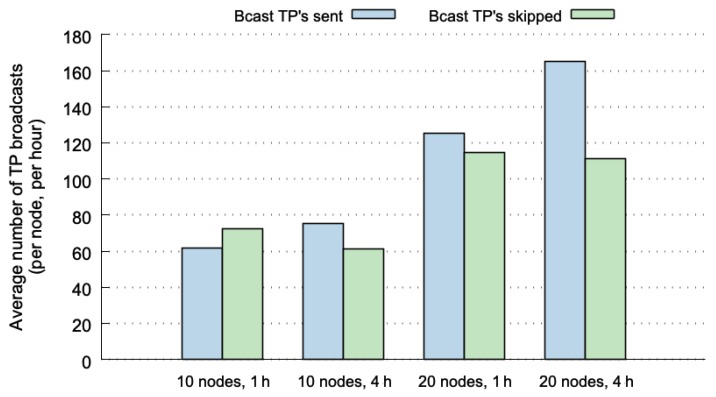
Average number of topology propagation (TP) broadcasts (per node, per hour).

Figure 5 plots the number of TP broadcasts for four different cases. The values shown in this figure were normalized, representing the average number of TP broadcasts per node and per hour. The values depicted in blue represent the number of broadcasts that are actually sent through the network. The broadcasts in green correspond to global hits, and therefore, to TP messages that are not transmitted (they can be correctly predicted at the destination nodes). The four cases used the SLAW mobility pattern considering 10 or 20 nodes, and 1 or 4 h of execution. The results show lower percentages of skipped TP broadcasts (between 40% and 54%). Although the values are normalized, we can observe that the cases with 20 nodes include more broadcasts because of both, the possible combinations of nodes, and the number of MPR nodes that broadcasts TP messages is higher.

If we compare the results shown in Figure 5 with those of the predictability limits reported in [12], the percentages of 70–85% of predictability limits for SLAW (considering 10, 20 nodes and 1, 4 h), decrease to 40–54% when using OLSR-HBP. This can be explained due to the fact that not all hits at origin nodes are global hits. Another explanation for this difference is that in Figure 5, the prediction percentages increased with larger historical-data windows, but OLSR-HBP performs better with shorter historical-data windows, as shown in [12]. This means that for this predictor it is more beneficial to work with shorter historical-data windows (using 1 h is better than 4 h). To take advantage of it, the predictor could delete the historical data every hour.

**Figure 6:**
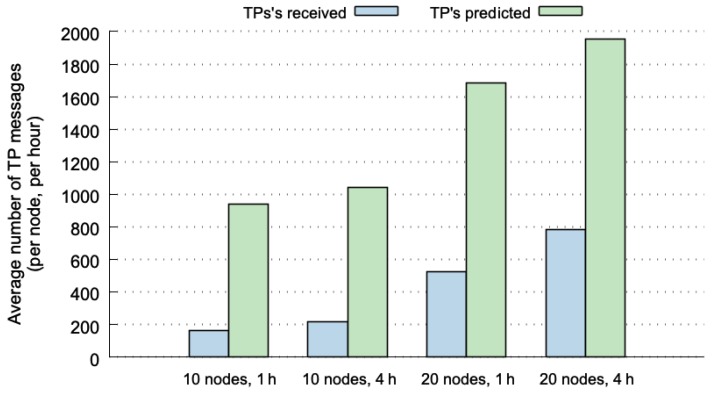
Average and normalized number of TP messages received/predicted at destination nodes.

Figure 6 shows the results from the perspective of the receiver of the TP messages. The absolute number of messages at the destination is greater than at origin (Figure 5) because every single broadcast message can be received by several nodes (we accumulate every TP message received at each node). The trends observed in Figure 5 are repeated now, but with higher percentages of predictable TP messages at the destination. In fact, SLAW with 10 nodes and 1 h historical-data window achieves 85.15% of prediction success. For the other cases, with the SLAW mobility pattern, the prediction percentage decreases with a higher number of nodes and larger historical-data windows. Particularly, it tends to decrease, reaching 82.83% (with 10 nodes and 4 h), 76.20% (with 20 nodes and 1 h) and 71.32% (with 20 nodes and 4 h).

#### 5.3.2. Memory Overhead

The last analysis conducted to determine the performance of OLSR-HBP was to compute the amount of memory occupation. The aim was to compute the amount of memory used by each node to store all the historical information needed by the predictor. OLSR-HBP basically uses two data structures: a vector to store the identifiers of the TP messages, and a table to store the HBP information (such as in Table 1). Each TP message contains an IPv4 address (4 bytes) to identify the originator node, and a list of the IPv4 addresses of its neighbour nodes. The greatest number of neighbours contained in a TP message was 8 (for 10 nodes cases) and 14 (for 20 nodes cases). The identifier assigned to each TP message requires 2 bytes. The table that contains the HBP information has entries with data for the pattern (a sequence of up to 5 identifiers), and the identifier of the most recent TP message received for this pattern. To compute the amount of memory used by OLSR-HBP, we did not consider the confidence bits nor the counters used by the most-frequent TP message algorithm.

**Figure 7:**
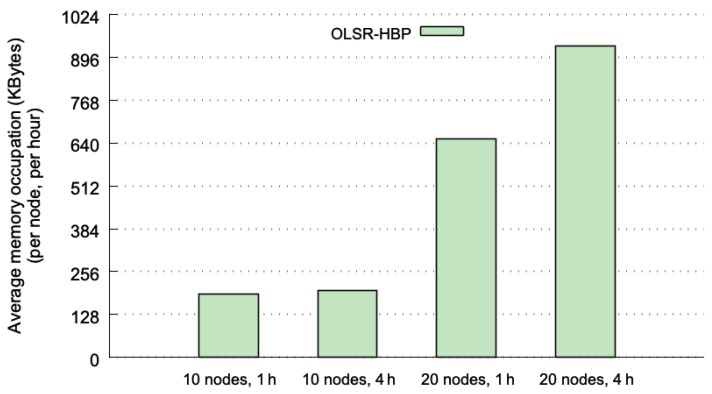
Average memory occupation (in KBytes) per node and per hour.

Figure 7 plots the average amount of memory (in KBytes) per node and per hour used, considering the four test cases. Each average value is computed from the same 40 executions as in the previous section. The grid 4 × 4 is the case with less memory requirements, because the number of different TP messages is very small. The memory used by OLSR-HBP in this last case is just about 128 KBytes (KB) per node.

The SLAW mobility pattern produces more variety in the TP messages, and therefore results in higher memory requirements. Such a mobility pattern involving 10 nodes needs 190 KB for the 1 h window, and 199 KB per hour for the 4 h window—it means a total of 796 KB for the whole execution (4 h). When the execution lasts 4 h, there is an increment of 4.8% in the memory requirements with respect to the case when the execution lasts 1 h. This slight increase may be caused by a greater variety of TP messages present when the execution lasts longer.

When the SLAW is used with 20 nodes, the average memory occupation increases to 652 KB (1 h window), and 930 KB per hour (4 h window; 3720 KB in total, a little less than 4 MBytes). These values represent an increase of 3.4 and 4.7 times for 1 h and 4 h executions respectively, compared to the values obtained with 10 nodes. This means that the memory occupation has a quadratic relationship with the number of nodes (when the number of nodes doubles, the memory occupation quadruples). The increase in the average memory used between 1 and 4 h is 24%. This value is larger than the previous 4.8% obtained for the 10 nodes cases. However, this increment is consistent with the quadratic increase observed in the number of nodes dimension. In summary, considering the worst case (20 nodes and 4 h), the amount of memory needed is 3.63 MBytes per node. This value is affordable for the memory capacities we can currently find on standard communication devices. Moreover, if the historical information is cleared every hour (as it is recommended to take into account the results of Section 5.3.1), the average memory requirements per node are reduced to just 420 KBytes. It is important to remark that the memory requirements of OLSR-HBP are much lower than those required by deep learning routing methods and its associated data structures [11,40,41,42].

### 5.4. Impact of OLSR-HBP on the Network Performance

In order to determine impact of the implemented predictor on the network, in this section we analyse the data transfer capability and the energy consumption of the network with and without OLSR-HBP. Next, we explain the evaluation performed and the obtained results.

#### 5.4.1. Data Transfer Capability

To confirm that OLSR-HBP does not negatively affect the data transmission through the network, we performed some additional tests. To simulate data traffic, we used periodic ping requests with 1 Kbyte payload each. We divided the network nodes into two groups. The first group sent periodic ping requests to one node in the second group (every pair of nodes transferred pings between them). The ping periods used were 10, 1 and 0.1 s. The analysed metrics were the message delivery rate (percentage of ping requests returned to the sender) and round-trip time (RTT—elapsed time between the instant when the request is sent and when the response is received), which corresponds to the latency. We compared the metric values between the original OLSR (where all TP messages are sent) and OLSR-HBP (where some TP messages were not sent, because they can be predicted at destination).

Table 7 shows the percentages of ping delivery (i.e., the number of pings received divided by number of pings sent). We consider the same four test cases of Section 5.3.1 for both, OLSR and OLSR-HBP. The first row represents the baseline values and corresponds to the delivery percentage of OLSR ($DP_{OLSR}$). The second row contains the delivery percentage ($DP_{OLSR-HBP}$) of the implemented predictor. Finally, the third row indicates the improvement achieved that is computed as $Improvement=\left(DP_{OLSR-HBP}-DP_{OLSR}\right)/DP_{OLSR}$. As a result, positive improvement values mean that OLSR-HBP achieves higher delivery percentage, whereas negative improvement values mean that the predictor performs worse than the original OLSR. We can also observe that OLSR-HBP performs similar to OLSR, with a maximum of −5.9% reduction in the delivery rate. This performance is reached when using a SLAW mobility pattern, with the highest number of nodes, largest execution time and highest ping frequency.

On the other hand, for the static/grid case, OLSR-HBP keeps the maximum delivery rate for a 10 s ping period. For a 1 s ping period, OLSR-HBP improves the delivery rate by 3.38%, and for a 0.1 s period its delivery rate is 7.23% lower than the regular OLSR protocol. The average improvement value is −2.28% (almost negligible), therefore, we can conclude that OLSR-HBP reaches and keeps the delivery rates of the regular OLSR protocol (baseline).

The second metric that we analysed was the average RTT (round-trip time) of the pings. Table 8 shows the RTT times (in milliseconds) for the four cases considered, and the percentage of improvement achieved. We denote as $RTT_{OLSR}$ and $RTT_{OLSR-HBP}$ to the RTT values for OLSR and OLSR-HBP respectively; then we define the RTT improvement as $RTT_{Improvement}=\left(RTT_{OLSR}-RTT_{OLSR-HBP}\right)/RTT_{OLSR}$. Due to the fact that the RTT is best for small values, $RTT_{Improvement}$ is positive when $RTT_{OLSR-HBP}$ is smaller than $RTT_{OLSR}$, and negative otherwise. The columns with ping periods of 0.1 s and execution times of 4 h could not be calculated given that the average RTT computation uses 16 bit sequences (icmp_seq), and at a rate of 0.1 s for 4 h, the number of pings overflows the 16 bit capacity. This means that the RTT computation is wrong.

For other cases where the RTT can be computed, we observe better values (less RTT) in 8 of 13 cases. Nevertheless, there are five cases where the RTT is worse for OLSR-HBP (particularly, all three grid cases). The differences (in percentage) among RTT range from 16.24% (good) to −18.9% (bad), with a global average of 0.50% that increases to 3.69% for SLAW mobility cases. After analysing these values in detail, it was not possible to determine the exact reasons for these variations, since they are probably caused by a combination of factors.

On the one hand, the decrease in the number of TP messages in the network releases communication resources that can be used to send data with less delay, and therefore with a good RTT. On the other hand, the loss of some TP messages or missed predictions at destination nodes may produce alterations in the routing information and configuration. Consequently, ping messages can be routed to a path with more hops, and therefore, it means larger RTT values.

#### 5.4.2. Energy Consumption

Another potential benefit of decreasing the number of TP messages sent through the network is the energy savings. In order to compute the reduction in the amount of energy consumed for transmissions and receptions, we used the NS-3 energy model WiFiRadioEnergy. In this model, we configured the transmitter and receiver electric current to 0.38 and 0.313 Amperes respectively. The rest of the parameters were set to 0 Amperes. Thus, we focus the energy analysis on the power needed to just send and receive data through the wireless network.

Table 9 shows the average normalized energy consumption for the four test cases. The values correspond to the average energy consumption (in Joules) per node and per hour, for the three ping periods considered. We performed 10 different executions for each of the three ping periods. The difference among the 10 executions was the initial location of the nodes and also the mobility paths, due to the impact of initial nodes location when the SLAW mobility pattern was used.

We can observe energy savings for the 10 s ping periods and 1 h execution times, specially in the 4 × 4 grid case (11.14% energy decrease). For the rest of the 1 h values, the energy consumption of OLSR-HBP increases up to 4.59% (2.76% in average) with regard to the regular OLSR protocol. When the execution time lasts 4 h, the normalized energy consumption increases in both, OLSR and OLSR-HBP. This increment is in average 16.15%, and it grows up to 30.0%. The higher energy consumption of 4 h versus 1 h cases, reinforces the previous conclusion that indicates the use of shorter execution times provide better prediction results for OLSR-HBP.

## 6. Conclusions and Future Work

This work presents the implementation of the OLSR-HBP, a TP message predictor that can be located between the routing and the network layers of mobile ad hoc networks. Its main goal is to reduce the number of TP messages that must be delivered through the network, and thus to decrease its congestion and energy consumption. Improving these aspects are particularly relevant in dynamic ad hoc collaboration scenarios, like responses to urban emergencies or disaster relief efforts, since it allows increasing the availability of the supporting systems. Typically, in these scenarios first responders (e.g., firefighters, police officers and medical personnel) count on weak and dynamic digital communication links, and the availability of these links depends on the battery available in the devices participating in the network. Reducing message congestion and energy consumption in the supporting networks usually improve the network availability and throughput, by easing thus the response process.

The performance of the OLSR-HBP predictor was evaluated involving communication scenarios with different complexity; all of them simulated urban emergencies and considered mobile ad hoc interactions among the nodes (e.g., first responders).

The evaluation results show that, for mobile networks with low density of nodes, the potential prediction capability of OLSR-HBP is around 80% when a TP message has already appeared in the past. This percentage falls to 50% when considering a mobile network with a higher node density. This analysis better explained that just a few messages contribute significantly to reducing the total percentage of TP messages delivered through the network. This represents a good opportunity to predict TP information, and this prediction can just be focused on a small subset of messages.

The implementation of the OLSR-HBP allows for the prediction of between 86% and 92% of the TP messages in static scenarios, and between 40% and 55% of them in high density mobile scenarios; which confirms the previous results. The network performance, in terms of throughput and latency, was similar to the original OLSR routing protocol, with −1.2% average throughput variations and −2.8% average latency variations. This means that the execution of the predictor did not affect the network operation.

The resources used by OLSR-HBP, in terms of energy and memory, were also evaluated in this study. The obtained results indicate that memory requirements of OLSR-HBP can be considered minimal, by taking into consideration the amount of memory resources available on current mobile devices. These low memory requirements confirm the suitability of OLSR-HBP to be implemented in mobile devices that act as communication nodes in mobile collaborative systems, like those supporting emergency responses.

In summary, the use of OLSR-HBP significantly decreases the signalling overhead, without disturbing the network operation, and requiring a small and affordable amount of resources. This prediction approach is deterministic and requires less computing resources than statistical approaches, such as machine or deep learning.

The next steps in this research effort will be focused on analysing the performance of this history-based prediction approach in ad hoc and opportunistic networks, and thus accurately establish the limits of this proposal. This further analysis will allow developers of mobile ad hoc systems and designers of routing protocols to understand how to take advantage of this prediction approach. The use of weighted functions to manage large historical data windows will be one of the approaches to be explored and analysed in detail.

## Figures and Tables

**Table 1:** Example of the history table for Node 1.

Pattern	Next	Count	Last
[2] [2]	[2]	3	
[2] [2]	[2,3]	1	✓
[2] [2,3]	[2,3]	1	✓
[2,3] [2,3]	[2,3]	3	
[2,3] [2,3]	[3]	1	✓
[2,3] [3]	[3]	1	✓
[3] [3]	[3]	3	✓

**Table 2:** OLSR-HBP simulation parameters.

Parameter	Values Used at Simulation
Node deployment	Static Grid 4 × 4
Surface/deployment area	120 × 120 m
Number of nodes	16
Confidence	0 bits (no confidence)
Prediction algorithm	Last Value
Data traffic	1 KByte periodic pings
Ping periods	no pings, 10 s, 1 s
NS-3 Parameter	Values used at simulation
wifiChannel	YansWifiChannelHelper
wifiChannel PropagationLoss	FriisPropagationLossModel
wifiPhy TxGain	−10
wifiPhy EnergyDetectionThreshold	−75.0

**Table 3:** Results of OLSR and OLSR-HBP in static scenarios.

	OLSR		OLSR-HBP	
Packet Generation Period	10 s	1 s	10 s	1 s
%TP msgs predicted @dest	-	-	99.9%	99.9%
Avg. % of pings received	100%	44.3%	+0%	+3.4%
Avg. RTT (ms)	11.6	50.7	−4.5%	−9.6%
Avg. Energy/node (J)	8.6	32.5	−11.5%	+4.1%

**Table 4:** Results of the performance metrics in static scenarios, using OLSR as ground truth.

Packet Generation Period	10 s	1 s
Precision (micro)	0.999	0.999
Precision (macro)	0.999	0.999
Recall (micro)	0.999	0.999
Recall (macro)	0.999	0.999
F1 (micro)	0.999	0.999
F1 (macro)	0.999	0.999
AUC (micro)	0.999	0.999

**Table 5:** Optimized link state routing (OLSR) history-based predictor (HBP) (OLSR-HBP) simulation parameters.

Parameter	Values Used at Simulation
Mobility pattern	SLAW
Surface/deployment area	300 × 300 m
Number of nodes	10, 20
Execution time	1 h, 4 h
Confidence	0 bits (no confidence)
Prediction algorithm	Last value
Data traffic	1 KByte periodic pings, from 1st to 2nd half of nodes
Ping periods	no pings, 10 s, 1 s, 0.1 s
NS-3 Parameter	Values used at simulation
wifiChannel	YansWifiChannelHelper
wifiChannel PropagationLoss	FriisPropagationLossModel
wifiPhy RxGain	−10
wifiPhy EnergyDetectionThreshold	−75.0

**Table 6:** Summary of classification performance metrics in mobile scenarios.

Packet Generation Period	-	10 s	1 s	0.1 s
Precision (micro)	0.767–0.897	0.768–0.897	0.767–0.897	0.767–0.899
Precision (macro)	0.719–0.842	0.719–0.842	0.717–0.839	0.715–0.851
Recall (micro)	0.767–0.897	0.768–0.897	0.767–0.897	0.768–0.899
Recall (macro)	0.723–0.838	0.722–0.837	0.721–0.834	0.720–0.845
F1 (micro)	0.767–0.897	0.768–0.897	0.767–0.897	0.768–0.899
F1 (macro)	0.720–0.839	0.720–0.839	0.718–0.836	0.717–0.847
AUC (micro)	0.896–0.948	0.897–0.948	0.896–0.948	0.897–0.948

**Table:** **a** SLAW 10 nodes 1 h

Ping Period	10 s	1 s	0.1 s
OLSR	18.2%	16.4%	16.2%
OLSR-HBP	18.1%	15.7%	16.0%
Improvement	−0.5%	−4.2%	−1.4%

**Table:** **b** SLAW 10 nodes 4 h

Ping Period	10 s	1 s	0.1 s
OLSR	22.8%	19.9%	19.2%
OLSR-HBP	22.7%	19.3%	19.1%
Improvement	−0.63%	−2.71%	−0.59%

**Table:** **c** SLAW 20 nodes 1 h

Ping Period	10 s	1 s	0.1 s
OLSR	23.5%	18.3%	18.3%
OLSR-HBP	22.6%	18.1%	17.3%
Improvement	−3.70%	−0.70%	−5.14%

**Table:** **d** SLAW 20 nodes 4 h

Ping Period	10 s	1 s	0.1 s
OLSR	32.3%	21.5%	21.2%
OLSR-HBP	31.0%	21.3%	19.9%
Improvement	−4.03%	−0.78%	−5.9%

**Table:** **a** SLAW 10 nodes 1 h

Ping Period	10 s	1 s	0.1 s
OLSR	14.3	8.51	6.97
OLSR-HBP	12.8	10.1	6.94
Improvement	10.5%	−18.9%	0.47%

**Table:** **b** SLAW 10 nodes 4 h

Ping Period	10 s	1 s
OLSR	11.7	11.5
OLSR-HBP	11.7	10.6
Improvement	0.02%	7.65%

**Table:** **c** SLAW 20 nodes 1 h

Ping Period	10 s	1 s	0.1 s
OLSR	12.6	20.4	12.5
OLSR-HBP	11.6	17.1	11.5
Improvement	7.93%	16.24%	8.61%

**Table:** **d** SLAW 20 nodes 4 h

Ping Period	10 s	1 s
OLSR	15.1	30.8
OLSR-HBP	13.8	32.0
Improvement	8.5%	−4.1%

**Table:** **a** SLAW 10 nodes 1 h

Ping Period	10 s	1 s	0.1 s
OLSR	2.01	6.34	51.4
OLSR-HBP	1.98	6.61	52.4
Improvement	1.5%	−4.3%	−2.0%

**Table:** **b** SLAW 10 nodes 4 h

Ping Period	10 s	1 s	0.1 s
OLSR	2.25	7.85	63.1
OLSR-HBP	2.33	8.57	70.7
Improvement	−3.6%	−9.1%	−12.1%

**Table:** **c** SLAW 20 nodes 1 h

Ping Period	10 s	1 s	0.1 s
OLSR	3.9	12.0	95.3
OLSR-HBP	3.84	12.3	97.7
Improvement	1.8%	−1.9%	−2.5%

**Table:** **d** SLAW 20 nodes 4 h

Ping Period	10 s	1 s	0.1 s
OLSR	5.30	17.5	142.6
OLSR-HBP	6.23	22.7	177.8
Improvement	−17.5%	−30.0%	−24.7%

## Appendix A. Individual OLSR-HBP Validation Metrics

**Table A1:** Classification performance metrics in mobile scenarios (10 nodes, 1 h HDWS, no confidence).

Packet Generation Period	-	10 s	1 s	0.1 s
Precision (micro)	0.875	0.875	0.876	0.876
Precision (macro)	0.826	0.826	0.829	0.833
Recall (micro)	0.875	0.875	0.876	0.876
Recall (macro)	0.813	0.814	0.816	0.823
F1 (micro)	0.875	0.876	0.876	0.876
F1 (macro)	0.818	0.819	0.822	0.827
AUC (micro)	0.938	0.937	0.938	0.938

**Table A2:** Classification performance metrics in mobile scenarios (10 nodes, 4 h HDWS, no confidence).

Packet Generation Period	-	10 s	1 s	0.1 s
Precision (micro)	0.860	0.860	0.860	0.860
Precision (macro)	0.803	0.800	0.797	0.800
Recall (micro)	0.860	0.860	0.860	0.860
Recall (macro)	0.802	0.800	0.797	0.802
F1 (micro)	0.860	0.860	0.860	0.860
F1 (macro)	0.802	0.800	0.796	0.800
AUC (micro)	0.930	0.930	0.930	0.930

**Table A3:** Classification performance metrics in mobile scenarios (10 nodes, 1 h HDWS, 2 bits conf).

Packet Generation Period	-	10 s	1 s	0.1 s
Precision (micro)	0.897	0.897	0.897	0.899
Precision (macro)	0.842	0.842	0.839	0.851
Recall (micro)	0.897	0.897	0.897	0.899
Recall (macro)	0.838	0.837	0.834	0.845
F1 (micro)	0.897	0.897	0.897	0.899
F1 (macro)	0.839	0.839	0.836	0.847
AUC (micro)	0.948	0.948	0.948	0.949

**Table A4:** Classification performance metrics in mobile scenarios (10 nodes, 4 h HDWS, 2 bits conf).

Packet Generation Period	-	10 s	1 s	0.1 s
Precision (micro)	0.879	0.879	0.880	0.881
Precision (macro)	0.819	0.825	0.831	0.827
Recall (micro)	0.879	0.879	0.880	0.881
Recall (macro)	0.824	0.828	0.836	0.832
F1 (micro)	0.879	0.879	0.880	0.881
F1 (macro)	0.820	0.825	0.833	0.829
AUC (micro)	0.939	0.939	0.940	0.940

**Table A5:** Classification performance metrics in mobile scenarios (20 nodes, 1 h HDWS, no confidence).

Packet Generation Period	-	10 s	1 s	0.1 s
Precision (micro)	0.798	0.798	0.798	0.799
Precision (macro)	0.734	0.734	0.737	0.740
Recall (micro)	0.798	0.798	0.798	0.799
Recall (macro)	0.723	0.725	0.727	0.728
F1 (micro)	0.798	0.798	0.798	0.799
F1 (macro)	0.727	0.729	0.732	0.733
AUC (micro)	0.899	0.899	0.899	0.900

**Table A6:** Classification performance metrics in mobile scenarios (20 nodes, 4 h HDWS, no confidence).

Packet Generation Period	-	10 s	1 s	0.1 s
Precision (micro)	0.767	0.768	0.767	0.767
Precision (macro)	0.719	0.719	0.717	0.715
Recall (micro)	0.767	0.768	0.767	0.768
Recall (macro)	0.723	0.722	0.721	0.720
F1 (micro)	0.767	0.768	0.767	0.768
F1 (macro)	0.720	0.720	0.718	0.717
AUC (micro)	0.896	0.897	0.896	0.897

**Table A7:** Classification performance metrics in mobile scenarios (20 nodes, 1 h HDWS, 2 bits conf).

Packet Generation Period	-	10 s	1 s	0.1 s
Precision (micro)	0.832	0.834	0.833	0.835
Precision (macro)	0.753	0.761	0.762	0.761
Recall (micro)	0.832	0.834	0.833	0.835
Recall (macro)	0.746	0.753	0.755	0.752
F1 (micro)	0.832	0.833	0.833	0.835
F1 (macro)	0.748	0.755	0.757	0.755
AUC (micro)	0.916	0.916	0.917	0.917

**Table A8:** Classification performance metrics in mobile scenarios (20 nodes, 4 h HDWS, 2 bits conf).

Packet Generation Period	-	10 s	1 s	0.1 s
Precision (micro)	0.790	0.790	0.792	0.793
Precision (macro)	0.738	0.735	0.736	0.737
Recall (micro)	0.790	0.790	0.792	0.793
Recall (macro)	0.742	0.739	0.740	0.743
F1 (micro)	0.790	0.790	0.792	0.793
F1 (macro)	0.739	0.736	0.737	0.739
AUC (micro)	0.907	0.906	0.906	0.907
